# Schistosomiasis and hepatopulmonary syndrome: the role of concomitant liver cirrhosis

**DOI:** 10.1590/0074-02760160383

**Published:** 2017-07

**Authors:** Liana Gonçalves-Macedo, Edmundo Pessoa Lopes, Ana Lucia Coutinho Domingues, Brivaldo Markman, Vitor Gomes Mota, Carlos Feitosa Luna

**Affiliations:** 1 Universidade Federal de Pernambuco, Programa de Pós-Graduação em Medicina Tropical, Recife, PE, Brasil; 2 Universidade Federal de Pernambuco, Departamento de Medicina Clínica, Serviço de Gastroenterologia e Hepatologia, Recife, PE, Brasil; 3 Universidade Federal de Pernambuco, Departamento de Medicina Clínica, Serviço de Cardiologia e Ecocardiografia, Recife, PE, Brasil; 4 Fundação Oswaldo Cruz-Fiocruz, Laboratório de Métodos Quantitativos em Saúde, Recife, PE, Brasil

**Keywords:** neglected diseases, portal hypertension, oesophageal and gastric varices, ultrasound, schistosomiasis, hepatopulmonary syndrome

## Abstract

**BACKGROUND:**

Hepatopulmonary syndrome (HPS) is defined as an oxygenation defect induced by intrapulmonary vasodilation in patients with liver disease or portal hypertension. It is investigated in patients with liver cirrhosis and less frequently in those with portal hypertension without liver cirrhosis, as may occur in hepatosplenic schistosomiasis (HSS).

**OBJECTIVES:**

To investigate the prevalence of HPS in patients with HSS, and to determine whether the occurrence of HPS is influenced by concomitant cirrhosis.

**METHODS:**

We evaluated patients with HSS with or without concomitant liver cirrhosis. All patients underwent laboratory testing, ultrasound, endoscopy, contrast echocardiography, and arterial blood gas analysis.

**FINDINGS:**

Of the 121 patients with HSS, 64 were also diagnosed with liver cirrhosis. HPS was diagnosed in 42 patients (35%) and was more frequent among patients with concomitant liver cirrhosis than in those without cirrhosis (42% vs. 26%), but the difference was not significant (p = 0.069). HPS was more common in those with spider naevi, Child-Pugh classes B or C and high model for end stage liver disease (MELD) scores (p < 0.05 each).

**MAIN CONCLUSIONS:**

The prevalence of HPS was 35% in this study. The occurrence of liver cirrhosis concomitantly with HSS may have influenced the frequency of patients presenting with HPS.

Hepatopulmonary syndrome (HPS) is defined as a clinical triad characterised by an oxygenation defect induced by the presence of pulmonary vasodilation observed in patients with liver disease or portal hypertension (Machicao & Fallon 2012). There are no pathognomonic signs or symptoms of HPS, although dyspnoea, platypnoea, peripheral cyanosis, digital clubbing, and spider naevi may be observed (Rodríguez-Roisin et al. 2004). While the pathophysiology of HPS has not been fully clarified, it is known that in portal hypertension, there is an increase in substances produced by the liver that could act on the lungs, thereby promoting vasodilation, with a consequent increase in the arterial alveolar oxygen gradient and hypoxemia (Machicao & Fallon 2012).

Portal hypertension may also lead to another vascular pulmonary complication, pulmonary arterial hypertension (Simonneau et al. 2013). Its pathophysiology is opposite to HPS, being pulmonary vasoconstriction and elevated pulmonary vascular resistance (Rodríguez-Roisin et al. 2004). Unlike HPS, pulmonary arterial hypertension may be idiopathic or secondary to various conditions besides portal hypertension (Simonneau et al. 2013). The diagnosis of pulmonary arterial hypertension is confirmed by haemodynamic studies, and occurs in 2% to 6% of patients with portal hypertension, including schistosomiasis (Lapa et al. 2009, Simonneau et al. 2013). The prevalence of HPS is lower in patients with non-cirrhotic portal hypertension (8% to 9.7%), than in those with cirrhosis (15% to 17.5%), in whom the presence of HPS is related to a poor prognosis (De et al. 2000, Martínez et al. 2001, Kaymakoglu et al. 2003, Fallon et al. 2008, Ferreira et al. 2008).

In Brazil, a leading cause of non-cirrhotic portal hypertension is hepatosplenic schistosomiasis (HSS), which results from chronic infection with *Schistosoma mansoni* (Gryseels 2012, Lambertucci 2014). When patients with HSS develop cirrhosis due to infection with hepatitis B virus (HBV) or hepatitis C virus (HCV), chronic alcohol consumption, or other causes, their prognosis worsens as a result of greater impairment of liver function, with a possibility of the involvement of other organs or systems (Bosch et al. 2008, Barsoum et al. 2013, Van-Lume et al. 2013).

In HSS patients, HPS is rarely reported, either because its prognostic implications are not well known, or because HSS patients typically show only mild impairment of their liver function and are therefore not candidates for liver transplantation (Ferreira et al. 2009, Lambertucci 2014). In addition, it has yet to be determined whether concomitant cirrhosis in HSS patients plays a role in the development of HPS. The objective of this study was to undertake a prospective investigation into the prevalence of HPS in patients with HSS, and to determine whether the occurrence of HPS is influenced by concomitant hepatic cirrhosis.

## SUBJECTS AND METHODS

This was a prospective, cross-sectional, hospital-based study, in which all patients diagnosed with HSS and oesophageal varices, with or without concomitant liver cirrhosis, between April 2010 and December 2012 were eligible for inclusion in this study protocol, i.e., screening for HPS. Splenectomised patients and those who had consumed alcohol in the past six months, presented with severe cardiac disease or severe restrictive or obstructive lung disorder observed on spirometry or were receiving antiviral therapy for hepatitis, were excluded.

Each patient was evaluated individually by the same observer. On this occasion, information regarding the period and place of exposure to *S. mansoni* was obtained through the patient’s origin and previous addresses, previous treatment with praziquantel (PZQ), previous blood transfusion, alcohol consumption and laboratory diagnosis of infection with HBV and HCV.

The diagnosis of HSS was based on a history of contact with water in an area endemic for *S. mansoni*, together with ultrasound findings of periportal hepatic fibrosis (Symmers’ fibrosis) and splenomegaly (Richter et al. 2001). The additional diagnosis of liver cirrhosis in patients with HSS was based on clinical findings, liver function test results, and ultrasound findings consistent with a diagnosis of liver cirrhosis (liver surface nodularity, evaluated by ultrasound at the left liver lobe) as well as on liver histology when a biopsy was performed (Bosch et al. 2008, Berzigotti et al. 2010). The severity of liver disease was assessed by Child-Pugh criteria (hepatic encephalopathy, ascites, prothrombin time or the international normalised ratio and serum levels of albumin and bilirubin) and the model for end-stage liver disease (MELD) score (Child and Turcotte 1964, Kamath et al. 2001). The severity of previous lung disease was assessed by performing forced spirometry in all patients (Micro Kit; Cosmed, Rome, Italy), and predicted values were based on the reference values for Brazil (Pereira et al. 2007).

The diagnosis of HPS was based on a widened alveolar-arterial partial pressure oxygen gradient (AaPO_2_) (≥ 15 mmHg or ≥ 20 mmHg for individuals over 64 years of age), with or without arterial hypoxemia (PaO_2_ < 80 mmHg), and intrapulmonary vascular dilatation (IPVD), as diagnosed by transthoracic contrast-enhanced echocardiography (Rodríguez-Roisin et al. 2004). Patients without HPS were included as controls.

*Procedures for diagnosing HPS*: *transthoracic contrast-enhanced echocardiography* - Echocardiography was performed with an ultrasound system (HDI 1500; Philips Medical Systems, Eindhoven, the Netherlands). With the patient in the lateral decubitus position, we punctured a vein in the arm and inserted a three-way catheter for contrast injection (0.9% saline). The test was considered positive for IPVD if opacification of the left atrium was observed between the fourth and the sixth cardiac cycles, counted from the opacification of the right atrium in the absence of intracardiac communications (Vedrinne et al. 1997).

*Arterial blood gas analysis* - Arterial blood samples were obtained through radial artery puncture while the patient was seated, at rest, and breathing ambient air at sea level. Each sample was immediately analysed (GEM 3000; Instrumentation Laboratory, Bedford, MA, USA) to determine the pH, PaO_2_ and arterial carbon dioxide tension (PaCO_2_) values. The AaPO_2_ value was calculated according to the alveolar gas equation (Rodríguez-Roisin et al. 2004).

*Statistical analysis -* Statistical analysis was performed using the Statistical Package for the Social Sciences, version 8.0 (SPSS Inc., Chicago, IL, USA). Continuous variables are expressed as mean ± standard deviation and categorical variables, as absolute and relative frequencies. As measures of association, we used the Student’s t-test for continuous variables and the *X* ^*2*^ test, together with Fisher’s exact test, when indicated, for categorical variables. The cases without HPS were used as controls. Values of p < 0.05 were considered statistically significant.

*Ethics -* The study was approved by the Research Ethics Committee of the Federal University of Pernambuco Health Sciences Centre (Protocol no. 396/2010). All participating patients provided written informed consent.

## RESULTS

Of the 175 patients diagnosed with HSS and oesophageal varices, 121 completed the study protocol. The study included 68 males (56.2%), and the mean age was 56 ± 12 years. All patients had been exposed to water contaminated by *S. mansoni*; 62 (51.2%) presented a history of treatment with PZQ, and 64 (52.8%) presented with the additional diagnosis of cirrhosis, the most common cause of which was alcohol consumption (43.8%) followed by HCV infection (28.1%) (Table I).

**TABLE I:** Age, gender and epidemiological characteristics of 121 patients with hepatosplenic schistosomiasis (HSS)

	N	(%)
Age (mean ± standard deviation)	55,6 ± 11,50	
Gender		
Male	68	56.2
Female	53	43.8
Exposure to *Schistosoma mansoni* contaminated water		
No	0	0.0
Yes	121	100.0
Previous treatment for schistosomiasis		
No	59	48.8
Yes	62	51.2
Hemotransfusion history		
No	74	61.2
Yes	47	38.8
Cause of cirrhosi^*^		
Alcohol	28	43.8
HCV	18	28.1
HBV-Alcohol	7	10.9
HBV	6	9.4
HCV-Alcohol	1	1.6
HCV-HBV	1	1.6
Cirrhosis through other causes	3	4.6

*: 64 patients with HSS with concomitant cirrhosis; HCV: hepatitis C virus; HBV: hepatitis B virus.

Forty-two patients (35%) were diagnosed with HPS. The prevalence of HPS was higher among patients with cirrhosis than among those without cirrhosis (42% vs. 26%, respectively), but the difference was not significant (p = 0.069). The prevalence of HPS was also higher among patients with spider naevi, those classified as Child-Pugh class B or C, and those with high MELD scores (p < 0.05 each) (Table II).

**TABLE II:** Clinical findings, arterial blood gas analysis and intrapulmonary vascular dilatation in hepatosplenic schistosomiasis (HSS) patients with and without hepatopulmonary syndrome (HPS)

	HPS (n = 42)	Non-HPS (n = 79)	p-value
Age (years), mean ± SD	52.79 ± 13.22	57.05 ± 10.25	0.055
Gender, n (%)			
Male	25 (60)	43 (54)	
Female	17 (40)	36 (46)	0.591
Clinical findings			
Liver disease, n (%)			
HSS	15 (36)	42 (53)	
HSS with cirrhosis	27 (64)	37 (47)	0.069
Spider naevi, n (%)	13 (31)	11 (14)	0.029
Child-Pugh class, n (%)			
A	23 (55)	64 (81)	
B/C	19 (45)	15 (19)	0.003
MELD score, mean ± SD	12.31 ± 3.68	10.46 ± 3.22	0.007
Spirometry			
FVC (% predicted)	76.90 ± 10.91	73.73 ± 14.10	0.173
FEV_1_(% predicted)	81.50 ± 10.80	78.20 ± 13.99	0.153
FEV_1_/ FVC	0.87 ± 0.07	0.86 ± 0.06	0.956
Arterial blood gas analysis			
PaO_2_, mean ± SD	77.60 ± 9.78	80.41 ± 11.35	0.177
PaCO_2_, mean ± SD	35.14 ± 3.94	36.67 ± 4.06	0.052
AaPO_2_^*^, mean ± SD	28.14 ± 9.51	23.42 ± 12.47	0.037
IPVD^*^, n (%)	42 (100)	9 (11)	< 0.001

IPVD: intrapulmonary vascular dilatations; MELD: model for end-stage liver disease; SD: standard deviation; *: diagnostic parameter for hepatopulmonary syndrome.

Regarding the spirometry parameters and arterial blood gas analysis parameters, there were no significant differences between the patients with and without HPS, with the exception of the AaPO_2_ value, which was higher in patients with HPS (p = 0.037) (Table II).

Regarding abdominal ultrasound parameters (Symmers’ fibrosis pattern, longitudinal diameter of the spleen and transverse diameters of the portal and splenic veins) and endoscopy parameters (calibre of the oesophageal varices and the presence of portal hypertensive gastropathy), we observed no significant differences between patients with and without HPS (Table III).

**TABLE III:** Ultrasound and endoscopic findings in hepatosplenic schistosomiasis (HSS) patients with and without hepatopulmonary syndrome (HPS)

	HPS (n = 42)	Non-HPS (n = 79)	p-value
Ultrasound findings			
Symmers’ Fibrosis Pattern, n (%)			
D	25 (60)	34 (43)	
E/F	17 (40)	45 (57)	0.086
Spleen, longitudinal diameter^*^	15.01 ± 2.24	15.81 ± 2.57	0.093
Portal vein, transverse diameter^*^	1.17 ± 0.31	1.24 ± 0.31	0.301
Splenic vein, transverse diameter^*^	0.95 ± 0.27	0.97 ± 0.31	0.790
Endoscopic findings			
Esophageal varices, n (%)			
Mild	24 (57)	45 (57)	
Medium/Large	18 (43)	34 (43)	0.985
Portal hypertensive gastropathy, n (%)	36 (86)	58 (73)	0.128

*: mean ± standard deviation.

## DISCUSSION

The main finding of this study was the high prevalence of HPS in patients with HSS, than in patient with non-cirrhotic portal hypertension and those with cirrhotic portal hypertension on liver transplant waiting lists, as that reported in other studies evaluating the same diagnostic arterial blood gas parameters (De et al. 2000, Martínez et al. 2001, Kaymakoglu et al. 2003, Ferreira et al. 2008). The prevalence of HPS observed in the present study was also higher than that reported in a study that examined patients with chronic forms of infection by *S. mansoni* (10% HPS prevalence) without mentioning the presence of oesophageal varices (Ferreira et al. 2009).

Although non-cirrhotic portal hypertension is associated with the development of HPS (Rodríguez-Roisin et al. 2004), it remains unclear whether the presence of HPS correlates with the highest levels of portal hypertension. In our study, parameters such as large-calibre oesophageal varices, portal hypertension gastropathy, very advanced periportal fibrosis, a greater longitudinal diameter of the spleen, portal collaterals and dilatation of portal vessels, usually present in patients with higher levels of portal hypertension (de Franchis & Dell’Era 2007, Andrade 2009, Berzigotti et al. 2013), were not significantly more frequent in patients with HPS. In fact, we observed that the prevalence of HPS was significantly higher among patients with spider naevi and high Child-Pugh and MELD scores, parameters that indicate the most severe liver function impairment. Thus, it is interesting to note that the incidence of HPS was higher in patients with concomitant cirrhosis than in those with HSS but without cirrhosis (42% vs. 26%), although there was only a trend of statistical significance.

These findings may suggest that, for patients in the present study, impaired hepatic function had a greater influence than the possible higher levels of portal pressure, over the occurrence of HPS. It may also be that the ultrasound and endoscopy changes secondary to portal hypertension, used herein as indirect parameters of portal hypertension, were insufficient to demonstrate any difference between patients with and without HPS. Hence, because the portal pressure was not measured, this point remains unclarified, and therefore constitutes one of the limitations of this study. However, the measurement of the hepatic venous pressure gradient, a method used to obtain the portal pressure level in patients with cirrhosis, does not properly assess the pressure in the venous portal system in patients with HSS because these patients present pre-sinusoidal portal hypertension (Coutinho 1968, Merkel & Montagnese 2011).

Another interesting finding of the present study was that the AaPO_2_ value was also elevated in patients without HPS. The involvement of lung tissue has been reported in patients with schistosomiasis, which was also described in an experimental study involving mice chronically infected with *S. mansoni* (de Faria et al. 1959, Fairfax et al. 2012). It should be noted that although the involvement of lung tissue was not a focus of our study, this might have contributed to the widened AaPO_2_, irrespective of the presence of IPVD, resulting in an overestimation of the frequency of HPS.

In conclusion, the concomitant occurrence of hepatic cirrhosis with HSS may have influenced the finding of a higher frequency of HPS in this study. Given that HPS interferes negatively in the prognosis of patients with cirrhosis, its investigation could be performed during the routine care of patients with HSS and oesophageal varices, especially in those patients with concomitant cirrhosis. The diagnostic evaluation of HPS in patients with schistosomiasis may require further evaluation of the additional involvement of interstitial lung disease in these patients. Further studies assessing the prognostic significance of HPS in patients with schistosomiasis would be of great interest to clinical practice.
